# Surface basicity mediated rapid and selective adsorptive removal of Congo red over nanocrystalline mesoporous CeO_2_†

**DOI:** 10.1039/d1na00412c

**Published:** 2021-09-21

**Authors:** Deepak Joshy, Seena Chakko, Yahya A. Ismail, Pradeepan Periyat

**Affiliations:** Department of Chemistry, University of Calicut Kerala India 673635 pperiyat@uoc.ac.in; Department of Environmental Studies, Kannur University Kerala India pperiyat@kannuruniv.ac.in

## Abstract

Herein we first report surface basicity mediated rapid and selective adsorptive removal of organic pollutants over nanocrystalline mesoporous CeO_2_. The role of surface features in controlling the selectivity and efficiency of adsorption is well known. Nevertheless, the possibility of tuning the adsorption capacity and selectivity of adsorbents through their surface characteristics remains less explored. In this work, the surface basicity of mesoporous CeO_2_ nanoparticles was improved by Er^3+^ doping under two different reaction conditions: *via* sol–gel and sol–hydrothermal methods. The nature and amount of surface basic sites were determined with the help of CO_2_ temperature programmed desorption (TPD). The adsorption capacity and selectivity of four different CeO_2_ samples were investigated using Congo red, methyl orange, and methylene blue as the model pollutants. From the adsorption studies, Er^3+^ doped CeO_2_ synthesized by the sol–gel method, having the highest amount of surface basic sites, proved to be the most efficient and highly selective adsorbent among the four developed variants of CeO_2_ towards Congo red. According to the proposed mechanism, surface basicity can be employed as a controlling parameter capable of tuning the adsorption capacity as well as the selectivity of CeO_2_ towards organic pollutants.

## Abstract 2

Surface basicity mediated rapid and selective adsorptive removal of organic pollutants over nanocrystalline mesoporous CeO_2_.

## Graphical abstract

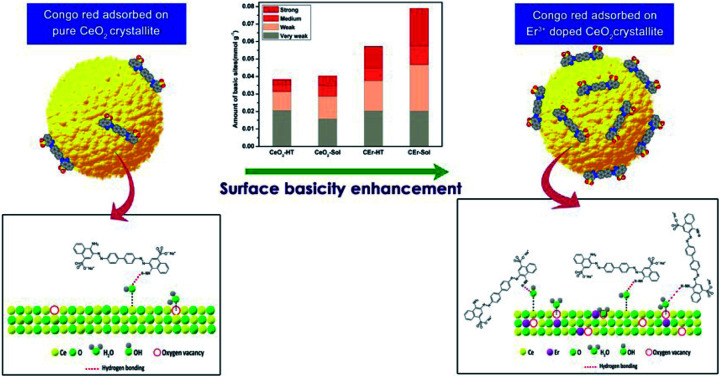

## Introduction

CeO_2_ is one of the most widely employed semiconducting metal oxides in the field of catalysis and environmental remediation,^1–5^ mainly due to (i) its high abundance and low cost,^6^ (ii) wide band-gap, non-toxicity and high stability,^7^ (iii) tendency for oxygen uptake into the lattice and the possibility of a reversible transition redox system between Ce^3+^ and Ce^4+^ (ref. 8 and 9) and (iv) the chance of formation of solid solutions with other oxides.^6^ CeO_2_ has already emerged as a promising choice for a wide range of catalytic processes such as a promoter in three-way catalysts in automobiles,^10,11^ solid oxide fuel cells,^12,13^ reforming of hydrocarbons,^14–16^ water gas shift reaction,^17–19^ CO oxidation,^20–22^ catalytic combustion of volatile organic compounds (VOC's),^23–26^ hydrogenation of alkynes,^27,28^ syngas conversion to alcohols,^29^ thermochemical water splitting,^30,31^ photocatalysis^32–34^*etc*. Nevertheless, efforts to further improve its catalytic efficiency are still in progress.^35^ Besides this, the environmental remediation applications of CeO_2_ mainly include photocatalytic degradation^36,37^ and adsorptive removal of pollutants from water resources.^38,39^

Textile and dyestuff industries are some of the major sources of water pollution, as they release dye species into water resources. The total world production of dyes is around 700 000 tonnes annually. About 10–15% of these dyes are lost during their application and a major share is discharged into water bodies. Many of these dyes have a very complex chemical structure and are found to be non-biodegradable. Studies have revealed that many of these dyes are carcinogenic and mutagenic in nature. In addition, the dyes may be present in different forms in different aqueous environments. In such cases we should be able to tune our remediation techniques according to the requirements of the target dye molecules. For example, Congo red is such a widely employed benzidine-based azo dye for various applications such as in textile, printing, plastic, rubber and dyeing industries. Due to its high water solubility, Congo red can disperse easily in water resources. Also depending on pH, Congo red is capable of being present in different ionic forms in water. Such a malign and widely distributed water pollutant should be treated individually by highly efficient means.^40–42^ Adsorptive removal is one such effective way to remove organic pollutants. While developing the adsorbent material, we have focused on Congo red as our target pollutant.

The adsorption capacity and selectivity of an adsorbent depend on several factors such as high surface area, porosity, amount of surface active sites, pH, electrostatic interaction between the adsorbent surface and dye species, and weak interactions such as hydrogen bonding between the adsorbent and dye molecules.^43,44^ The adsorption capacity and selectivity of adsorbents can be controlled by the effective tuning of the above factors particularly by regulating surface features. Hence in this study, we targeted the tailoring of the surface features of the adsorbent according to the requirement of the pollutant.

The present work aims at understanding, analysing and correlating the surface characteristics of CeO_2_ with its adsorption capacity and selectivity towards Congo red. The surface characteristics under investigation are surface area, porosity, surface basicity and hydrogen bonding. Variations in surface characteristics were brought by doping Er^3+^ into the CeO_2_ lattice under two different reaction conditions: *via* sol–gel and sol–hydrothermal methods. Er^3+^ doping succeeded in improving surface features such as surface area, porosity, surface basicity and thereby the weak interactions between the CeO_2_ surface and Congo red molecules. The effect of improved surface features was then correlated with the adsorption ability of CeO_2_. In this work, surface features of CeO_2_ were tuned to develop highly efficient and selective adsorbents for Congo red adsorption and removal. This work aims at maximizing the adsorption efficiency and selectivity of CeO_2_ with minimum modifications. This work will prompt future investigators to see adsorption, also from the perspective of surface basicity.

## Experimental

### Material preparation

Pure CeO_2_ and Er^3+^ doped CeO_2_ were synthesized by two separate synthetic routes *via* sol–hydrothermal and aqueous sol–gel methods. 43.2 g of Ce(NO_3_)_3_·6H_2_O (99%, Aldrich) was stirred in 500 mL distilled water for half an hour to ensure complete dissolution. NH_4_OH (Merck Emplura, 25%) solution was then added dropwise to precipitate cerium(iv) hydroxide. The addition of NH_4_OH was continued until the pH reached a value of 10 to ensure that all Ce(NO_3_)_3_·6H_2_O had been precipitated as Ce(OH)_4_. The precipitate of Ce(OH)_4_ was centrifuged and washed several times with distilled water. To confirm the absence of nitrate in the precipitate, concentrated H_2_SO_4_ was added to the centrifugate and the resulting solution was boiled. In this solution, a paper ball was dropped, and the absence of brown fumes indicated that the centrifugate was nitrate-free. After achieving the nitrate-free centrifugate, the precipitate was then dispersed in 1000 mL distilled water; to this, 10% HCl (Emplura Merck, 35%) was added dropwise until the pH value reached 2. The solution was then kept under stirring for 2 days to obtain the sol. The sol was then divided into two portions. The first portion was used for the synthesis of CeO_2_*via* the hydrothermal method. For this, the sol was transferred to a Teflon lined stainless steel autoclave and heated at 150 °C for 48 hours. The components in the autoclave were then transferred to a Petri dish and were dried in an oven set at 150 °C for 2 days to obtain hydrothermally synthesised CeO_2_ (CeO_2_-HT). The second portion of the sol was used for the synthesis of CeO_2_ by the aqueous sol–gel method. The sol was dried directly in an oven set at 150 °C for 48 hours. The dried precursor was then calcined at 500 °C for 2 hours and the compound thus obtained is represented as CeO_2_-Sol. The Er^3+^ doped CeO_2_ sol was prepared by the same procedure by adding a calculated quantity (5 mmol) of Er(NO_3_)_3_·5H_2_O to 100 mmol CeO_2_ sol. The Er^3+^ doped sol was subjected to both hydrothermal and sol–gel methods to obtain CEr-HT and CEr-Sol samples.

### Characterisation of materials

The phase purity and crystal structure of the synthesised samples were determined by powder X-ray diffraction (PXRD) on a Rigaku Miniflex 600 X-ray diffractometer. The crystallite size of the synthesised compounds was calculated from the PXRD pattern using the Scherrer equation. The surface characterisation and morphology evaluation were carried out using a ZEISS Gemini SEM 300. HR-TEM analysis was performed using a Jeol-JEM 2100 transmission electron microscope. Fourier transform infrared (FTIR) spectra measurements were carried out using a Jasco-FT/IR-4100 spectrophotometer. The Brunauer–Emmett–Teller (BET) surface area of the samples was calculated by N_2_ adsorption at the temperature of liquid nitrogen using a Belsorp Max surface area analyser. Prior to the measurements, the samples were degassed at 200 °C under vacuum for 3 hours to remove the adsorbed moisture on the catalyst surface. The specific surface area was calculated using the BET model at a relative pressure of *P*/*P*_0_ = 0.05–0.3.

### Basicity measurements using temperature programmed desorption (TPD)

CO_2_-temperature programmed desorption (TPD) studies were carried out using a BELCAT-M analyser. For this, 0.1 g of the prepared sample was weighed into a quartz tube sample holder and then subjected to pre-treatment at 200 °C for 30 minutes under a He atmosphere. The sample was then cooled to room temperature and then CO_2_ was passed over the sample for 30 minutes to carry out adsorption. Then it was followed by He purging for another 30 minutes at 50 °C for the removal of physisorbed CO_2_ from the sample surface. The desorption measurements were performed by increasing the temperature from 50 °C to 650 °C at a heating rate of 12 K min^−1^. The amounts of different types of basic sites were calculated by integrating the CO_2_-TPD curves over different temperature ranges of desorption corresponding to very weak, weak, medium and strong basic sites.

### Adsorption experiments

Adsorption studies were carried out on all four synthesized CeO_2_ samples (1 g L^−1^) using Congo red as the model pollutant at a concentration of 20 mg L^−1^. The adsorption experiments were carried out in magnetically stirred glass vessels at the ambient pH of the Congo red solution. At regular contact intervals, samples were withdrawn, centrifuged and analysed using a Jasco V-770 UV-vis-NIR spectrophotometer. To evaluate the selectivity, adsorption analyses were performed with methylene blue and methyl orange also. The effect of dye concentration and pH on adsorption activity was evaluated by varying the initial dye concentrations (10, 15, 20, 25 and 30 mg L^−1^) and by carrying out adsorption studies under 3 different pH conditions (3, 6.5 (ambient pH) and 10). Besides this, the pH of the point of zero charge (pH_PZC_) of the adsorbent material was determined using the pH drift method.^45^

### Adsorbent regeneration and reusability

After the adsorption process, the Congo red adsorbed CeO_2_ samples were collected and washed several times with distilled water. Then the samples were dried and calcined at 500 °C for 2 hours. The adsorption efficiency of the recycled adsorbents was also determined.

## Results and discussion

**Fig. 1:**
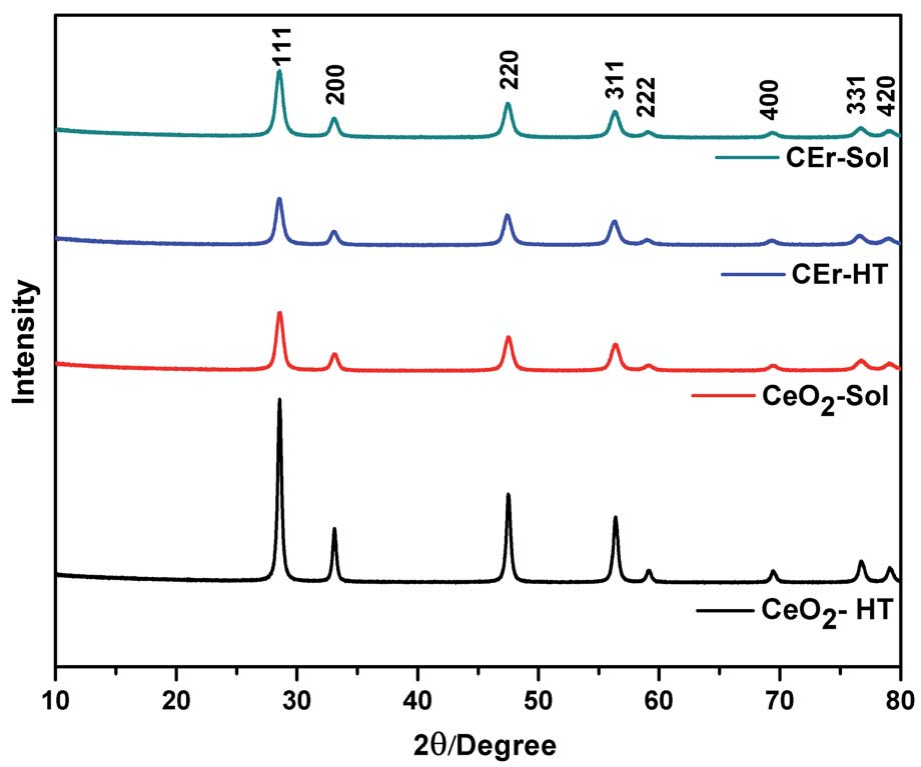
X-ray diffraction patterns of pure CeO_2_ and Er^3+^ doped CeO_2_ samples prepared by aqueous sol–gel and hydrothermal methods.

Pure CeO_2_ and Er^3+^ doped CeO_2_ were synthesised *via* hydrothermal and aqueous sol–gel methods. The PXRD patterns of all the samples synthesised are shown in Fig. 1. As shown in Fig. 1, well-defined peaks are obtained for all samples.

The peaks can be indexed to the cubic fluorite structure of CeO_2_ (JCPDS 34-0394) belonging to the *Fm*3*m* space group.^7^ Characteristic reflections of the (111), (200), (220), (311), (222) and (400) planes are shown in Fig. 1. A small shift in the peaks towards lower 2*θ* for Er^3+^ doped samples was observed in the PXRD pattern, and this can be attributed to the increased ionic size of Er^3+^ compared to that of Ce^4+^ (ionic radius of Ce = 97 pm; Er = 100.4 pm).

The crystallite sizes of the synthesised samples determined from the Scherrer equation are reported in Table 1. From the crystallite size values, it is evident that CeO_2_-HT has the largest crystallite size compared to other samples. This is due to the enhanced Ostwald ripening and oriented attachment in hydrothermally synthesised samples.^46^ However, in the case of CEr-HT, a reduction in the crystallite size was observed which may be due to the inhibition of crystal growth caused by the dopant Er^3+^. The presence of Er^3+^ in between the Ce^4+^ ions has a significant role in decreasing the frequency of collisions between the ceria particles. As the collisions decrease, the oriented attachment and Ostwald ripening rate diminish, resulting in a smaller crystallite size. At the same time, crystal growth takes place in a normal manner in the case of CeO_2_-Sol and CEr-Sol where strict conditions of pressure and temperature are absent. Thus, the crystal growth conditions are almost the same for CeO_2_-Sol and CEr-Sol.

**Table 1:** Calculated crystallite sizes of pure CeO_2_ and Er^3+^ doped CeO_2_ samples prepared by sol–gel and hydrothermal methods

Samples	Crystallite size (nm)
CeO_2_-HT	19.48
CeO_2_-Sol	10.74
CEr-HT	10.69
CEr-Sol	11.11

**Fig. 2:**
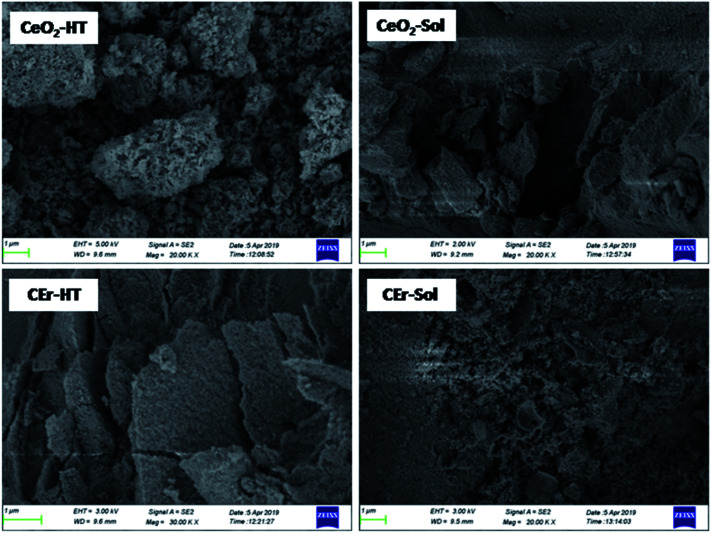
FESEM micrographs of pure CeO_2_ and Er^3+^ doped CeO_2_ samples prepared by sol–gel and hydrothermal methods.

The field emission scanning electron microscopy (FESEM) micrographs of the synthesized samples are shown in Fig. 2. An idea about the surface morphology and extent of agglomeration can be obtained from the FESEM images. The SEM images of CeO_2_-HT show large-sized aggregates, distinct from one another. At the same time, CEr-HT exhibits a porous flake like structure. The SEM images of pure CeO_2_ and Er^3+^ doped CeO_2_ particles synthesised *via* the sol–gel method indicate that CEr-sol exhibits a highly porous appearance and relatively small agglomerates when compared to CeO_2_-Sol.

**Fig. 3:**
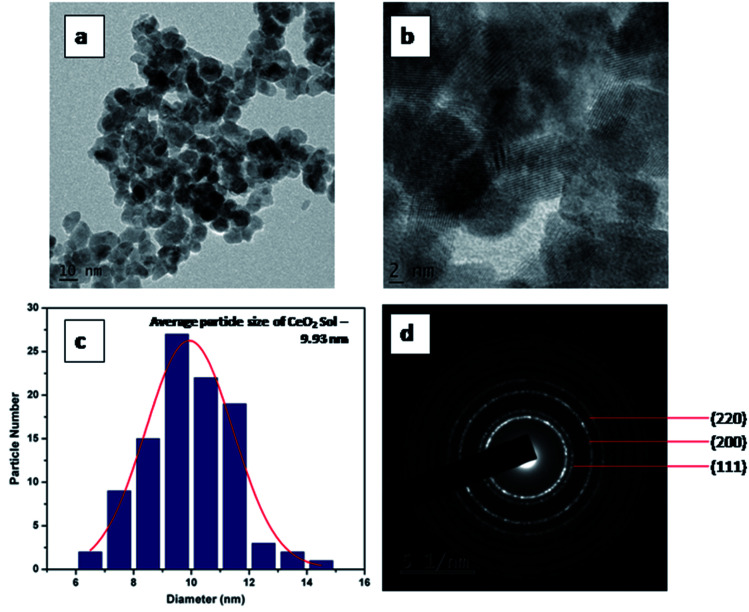
(a and b) HR-TEM images, (c) particle size distribution and (d) SAED pattern of CeO_2_-Sol.

HR-TEM analyses of CeO_2_-Sol and CEr-Sol were performed to further confirm the trend observed in crystallite sizes. HR-TEM images of CeO_2_-sol are shown in Fig. 3. The average particle size was found to be 9.93 nm. The SAED patterns can be indexed to (111), (200) and (220) planes. The calculated *d* spacing for the (111) plane is 3.14 Å in CeO_2_-Sol.

**Fig. 4:**
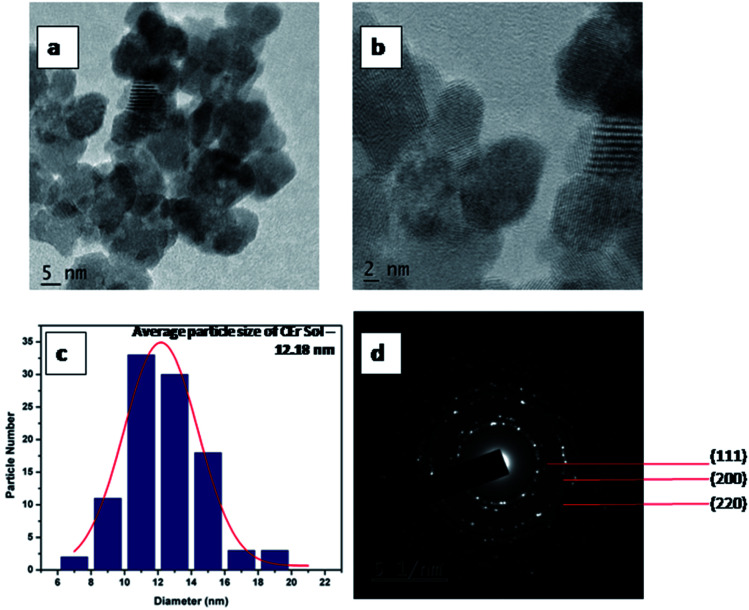
(a and b) HR-TEM images, (c) particle size distribution and (d) SAED pattern of CEr-Sol.

HR-TEM images of CEr-Sol are shown in Fig. 4. In this case, the average particle size was found to be 12.18 nm. Here the calculated *d* spacing for the (111) plane is 3.20 Å which is greater than that observed in CeO_2_-Sol. This can be attributed to the doping of relatively large Er^3+^ ions into the CeO_2_ lattice. The well defined fringes in Fig. 3b and 4b show the mesoporous nature of the prepared CeO_2_ and it is also well evident from the pore size obtained from the BET measurements.

**Fig. 5:**
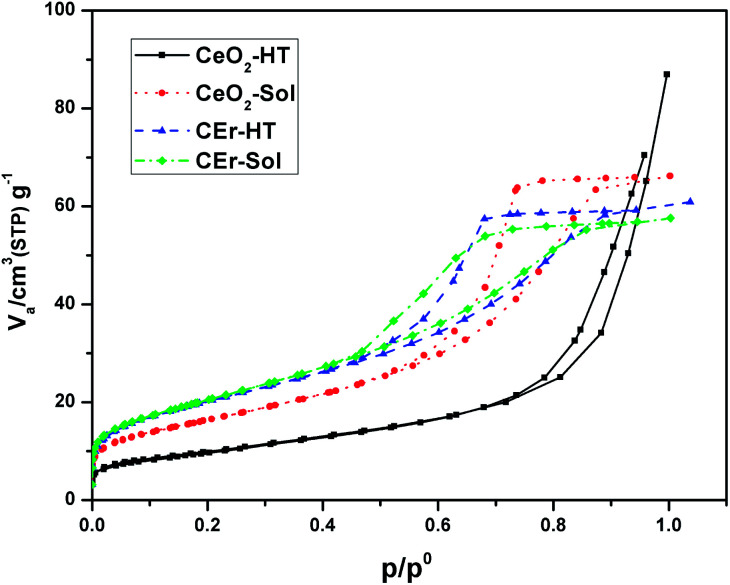
N_2_ adsorption isotherms of pure and Er^3+^ doped CeO_2_ samples prepared by aqueous sol–gel and hydrothermal methods.

The N_2_ adsorption isotherms of the synthesised samples are shown in Fig. 5. Parameters such as surface area, pore volume and pore diameters of the synthesised samples were analysed using the BET technique and are tabulated in Table 2. The Er^3+^ doped CeO_2_ samples were found to have a higher surface area than pure CeO_2_. Among the samples synthesised, the sol–gel derived samples exhibited higher surface areas than their hydrothermal analogues. In the case of hydrothermally synthesized samples, CEr-HT exhibited almost double the surface area of CeO_2_-HT. This can be partly correlated to the larger crystallite size of CeO_2_-HT particles which may result in a decrease of the surface area. The additional enhancement in surface areas of the Er^3+^ doped samples can be the result of the generated oxygen vacancies and the enriched interconnected pore networks present in them. All the samples synthesised were mesoporous in nature as evident from their pore size which lies in the range of 4–14 nm.^47^ The mesoporous nature is also evident from the fact that all the samples exhibit type IV adsorption isotherms.^48^ According to the IUPAC classification of adsorption hysteresis loops, the loops of CeO_2_-Sol, CEr-Sol and CEr-HT belong to type H2,^49^ which arises from porous materials having networks of interconnected pores of progressive sizes and shapes. At the same time, CeO_2_-HT exhibits a type H3 adsorption hysteresis loop which is characteristic of materials with slit-shaped pores.^50^

**Table 2:** Surface area parameters of pure CeO_2_and Er^3+^ doped CeO_2_ samples prepared by aqueous sol–gel and hydrothermal methods

Samples	BET surface area (m^2^ g^−1^)	Total pore volume (cm^3^ g^−1^)	Pore diameter (nm)
CeO_2_-HT	35.114	0.1278	14.563
CeO_2_-Sol	58.769	0.102	6.9446
CEr-HT	72.073	0.0929	5.1561
CEr-Sol	73.668	0.0888	4.8198

**Fig. 6:**
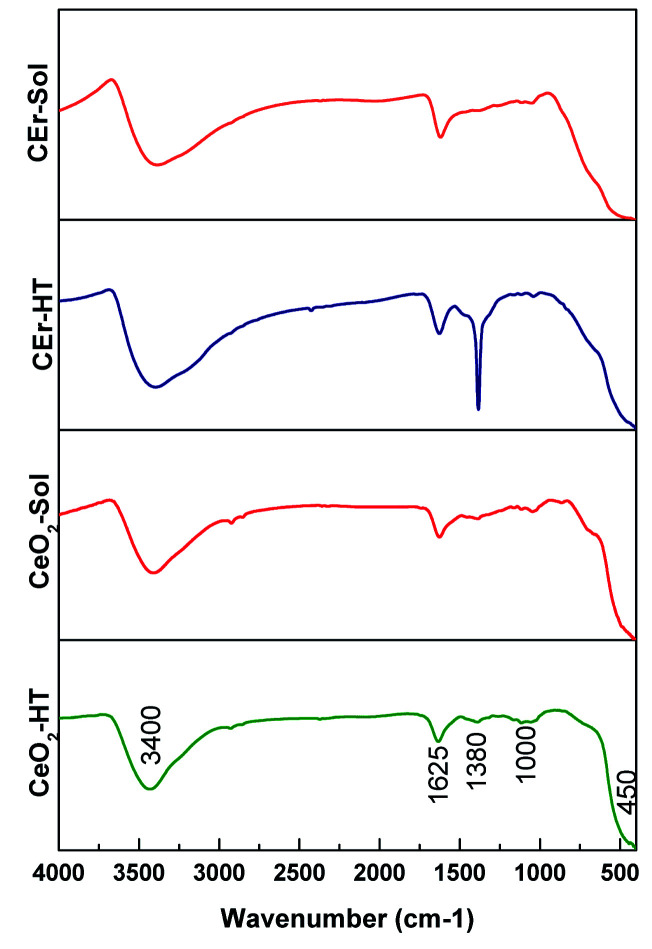
FT-IR spectra of pure and Er^3+^ doped CeO_2_ samples prepared by sol–gel and hydrothermal methods.

Here we have recorded the FT-IR spectra of all the CeO_2_ samples prepared and the spectra are shown in Fig. 6. In the FT-IR spectra, the broad absorption band within the range of 3400–3450 cm^−1^ corresponds to the OH stretching vibrations of adsorbed H_2_O on the sample surfaces.^51^ Again the transmission bands at 1380 and 1625 cm^−1^ correspond to the H–O–H bending mode which confirms the presence of adsorbed moisture and surface hydroxyls in the samples. Thus FT-IR measurements indicated the presence of adsorbed water molecules on the CeO_2_ surfaces. The role of adsorbed water in the selective adsorption of Congo red by CeO_2_ will be discussed later. The absorption bands at 450 cm^−1^ and 850 cm^−1^ correspond to characteristic Ce–O stretching vibrations. So the observed IR absorption frequencies are in good agreement with previous literature.^52,53^ Another species of strong band is located around 1000 cm^−1^, which may be associated with the formation of nano-crystalline CeO_2_.^52^

**Fig. 7:**
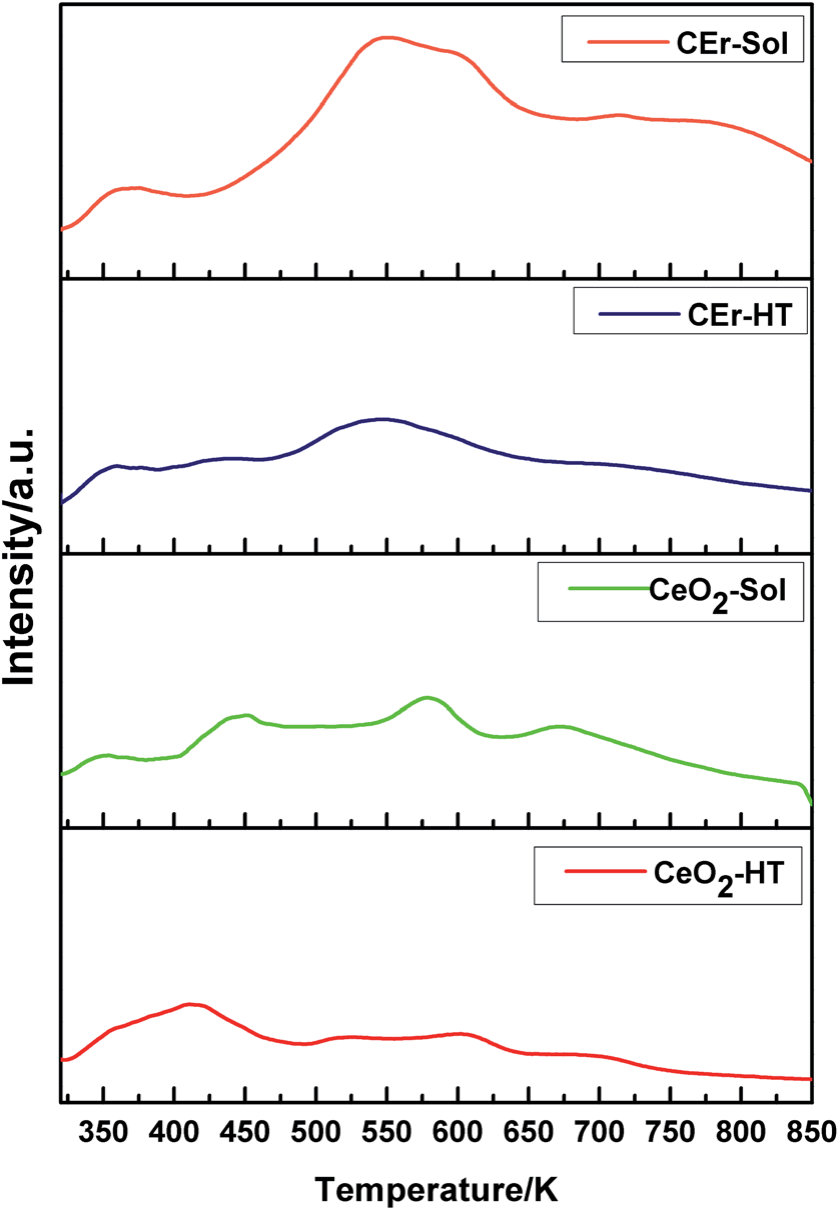
CO_2_-TPD curves at a heating rate of 12 K min^−1^ for CeO_2_-HT, CeO_2_-Sol, CEr-HT and CEr-Sol samples.

Surface basicity studies performed using CO_2_-TPD measurements revealed the strength, distribution and amount of basic sites present on the surface of the synthesised compounds.^54,55^ The strength of basic sites was determined based on the temperature range in which desorption occurs *i.e.*, the higher the temperature at which desorption occurs, the stronger will be the basic sites. Similarly, weaker sites desorb at a lower temperature. Based on the temperature range at which desorption occurs, the basic sites on CeO_2_ particles were classified into very weak (<523 K), weak (523–653 K), medium (653–723 K) and strong (>723 K).^56,57^ Integration of the CO_2_-TPD curves over these temperature ranges provided the amount of different basic sites. The TPD curves of all the samples recorded at a heating rate of 12 K min^−1^ are shown in Fig. 7 and the amount of basic sites calculated by integrating the curves and the surface area of the respective samples are given in Table 3.

**Table 3:** Surface area, amount of different basic sites and the total amount of basic sites for CeO_2_-HT, CeO_2_-Sol, CEr-HT and CEr-Sol samples

Sample	Surface area (m^2^ g^−1^)	Type of basic site				Total (mmol g^−1^)
		Very weak < 523K (mmol g^−1^)	Weak 523–653 K (mmol g^−1^)	Medium 653–723 K (mmol g^−1^)	Strong > 723 K (mmol g^−1^)	
CeO_2_-HT	35.11	0.0205	0.0107	0.0038	0.0034	0.0384
CeO_2_-Sol	58.77	0.0157	0.0130	0.0059	0.0058	0.0404
CEr-HT	72.07	0.0203	0.0173	0.00687	0.0128	0.0573
CEr-Sol	73.67	0.0202	0.0265	0.0104	0.0219	0.0790

**Fig. 8:**
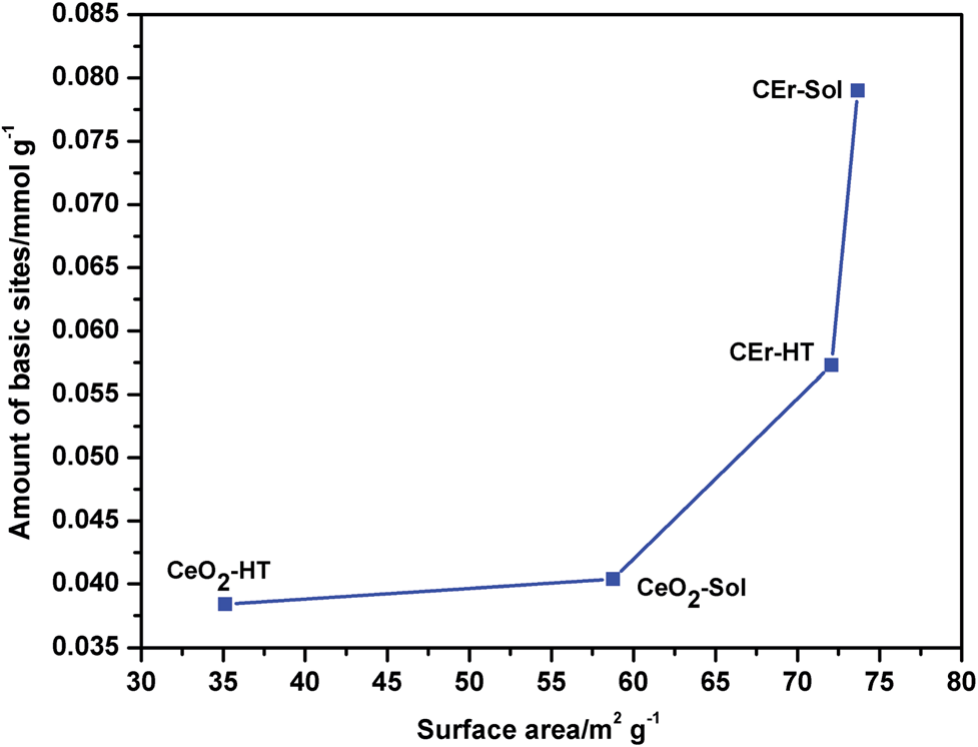
Variation in the amount of basic sites *vs* surface area of various CeO_2_ samples prepared.

**Fig. 9:**
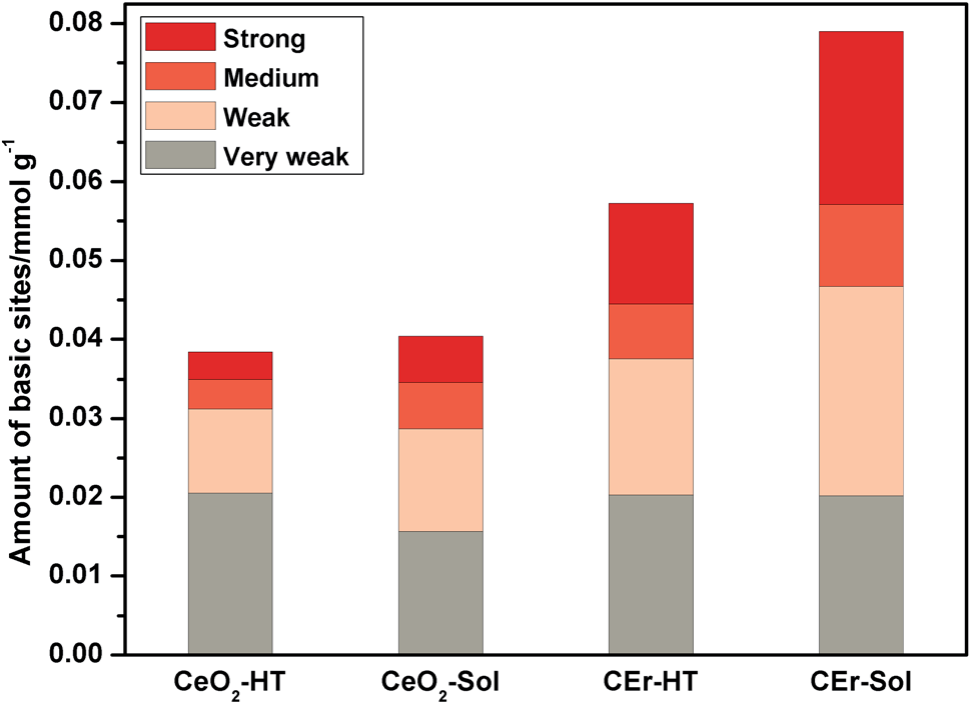
Distribution of different basic sites in various CeO_2_ samples.

It is evident from Fig. 8 that with the increase in the surface area there is a simultaneous increment in the number of basic sites. While considering each type of basic site, except for the very weak type sites, the amount of all other types of basic sites increases with an increase in surface area. However, very weak basic sites are present in almost the same amount in all samples except in CeO_2_-Sol. The distributions of different types of basic sites among the synthesised samples are shown in Fig. 9.

The introduction of Er^3+^ ions into the CeO_2_ lattice has a remarkable influence on the strength of basic sites. For hydrothermally synthesized samples (CeO_2_-HT and CEr-HT), the amount of medium strength basic sites got doubled on Er^3+^ doping and the amount of strong basic sites became four times that of CeO_2_-HT. Similarly, in sol–gel synthesized samples, CEr-Sol has almost double the amount of medium strength basic sites and approximately four times the amount of strong basic sites when compared to CeO_2_-Sol.

**Fig. 10:**
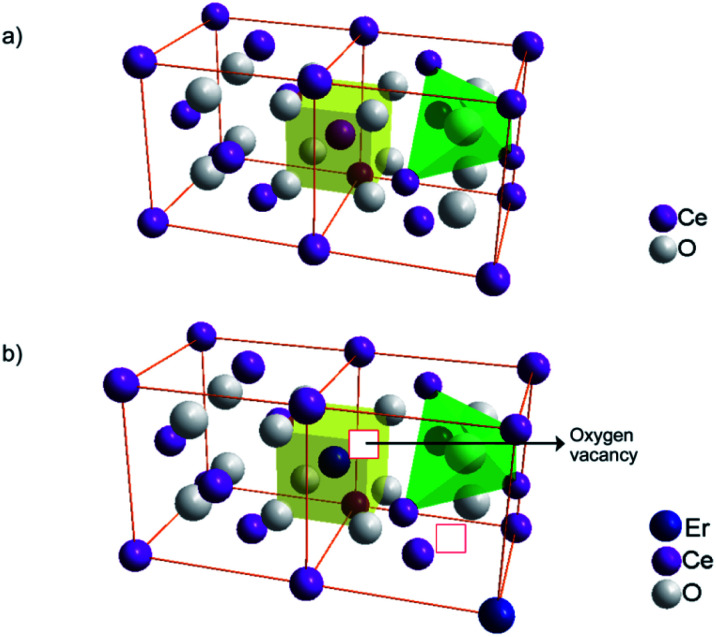
Structure of (a) pure CeO_2_ and (b) Er^3+^ doped CeO_2_ with oxygen vacancies in the lattice.

The increase in the number of basic sites with Er^3+^ doping can be explained based on the Lewis acid–base concept. The structure of pure CeO_2_ and oxygen vacancy generation by Er^3+^ doping in the CeO_2_ lattice are illustrated in Fig. 10. The replacement of Ce^4+^ with Er^3+^ in the CeO_2_ lattice results in oxygen vacancies, which are electron-rich in nature. According to the Lewis concept, electron donors are basic in nature and electron acceptors are acidic. Hence the electron-rich sites are expected to have basic character.^58^ So, the doping of Er^3+^ results in an increased number of oxygen vacancies which are basic in nature.

### Adsorption studies

**Fig. 11:**
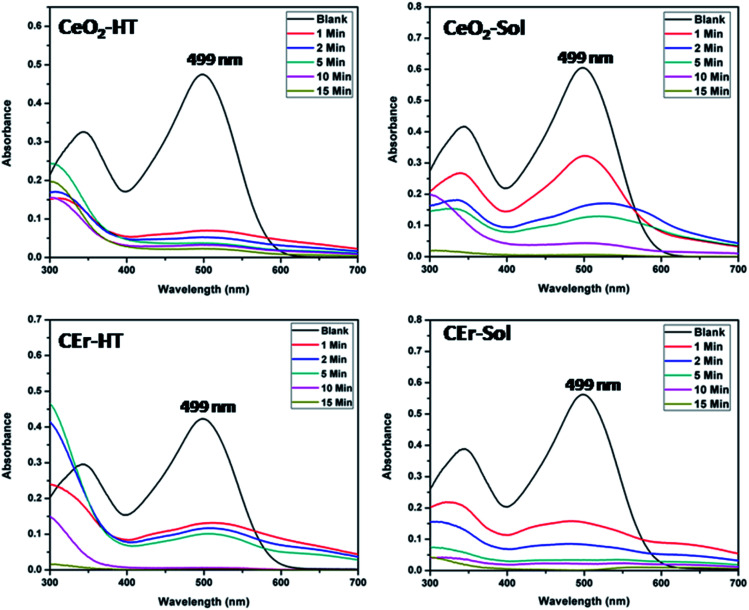
UV-visible absorbance spectra showing the adsorptive removal of Congo red by CeO_2_-HT, CeO_2_-Sol, CEr-HT and CEr-Sol samples.

The adsorption activity of the as-prepared CeO_2_ samples towards Congo red was evaluated using UV-visible absorption spectroscopy. The adsorptive removal of Congo red (initial concentration of the dye solution was fixed at 20 mg L^−1^) by the CeO_2_-HT, CeO_2_-Sol, CEr-HT and CEr-Sol samples (1 g L^−1^) at the ambient pH (6.5) of Congo red solution in terms of their UV-visible spectra is shown in Fig. 11.

**Fig. 12:**
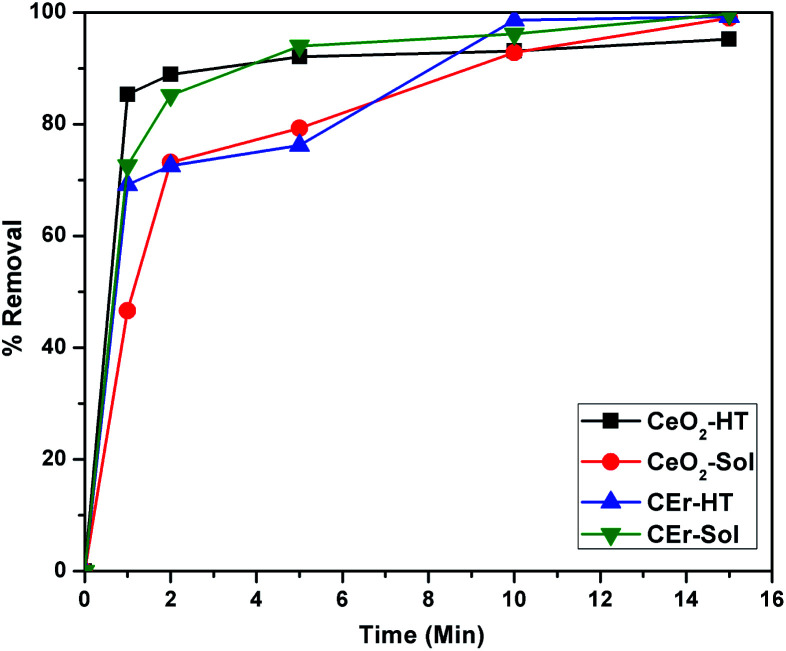
Removal percentages of Congo red by CeO_2_-HT, CeO_2_-Sol, CEr-HT and CEr-Sol samples.

**Fig. 13:**
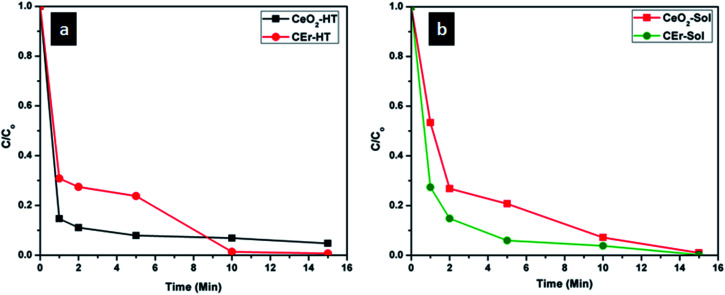
Comparison of adsorption rates of (a) sol–hydrothermal derived and (b) sol–gel derived CeO_2_ samples.

The percentage removal of Congo red by the four different CeO_2_ samples is shown in Fig. 12. It can be seen that all four samples show more than 90% removal of Congo red within 15 minutes. It can be seen that CeO_2_-HT and CEr-Sol show 88.9 and 85.16% removal within 2 minutes. Among the four samples, CEr-Sol is capable of removing almost 100% of Congo red within 15 minutes. The adsorption rates of Congo red by the sol–hydrothermal and sol–gel derived samples are shown separately in Fig. 13. In the case of hydrothermal derived samples, it can be seen that CeO_2_-HT initially shows a higher rate of adsorption which diminishes later and then CEr-HT dominates. While comparing the sol–gel derived samples, it can be seen that CEr-Sol always exhibits a superior adsorption rate to CeO_2_-Sol. From the adsorption studies, it is evident that CEr-Sol is the best adsorbent material among the four CeO_2_ samples developed. Therefore further investigations were focussed mainly on CEr-Sol.

**Fig. 14:**
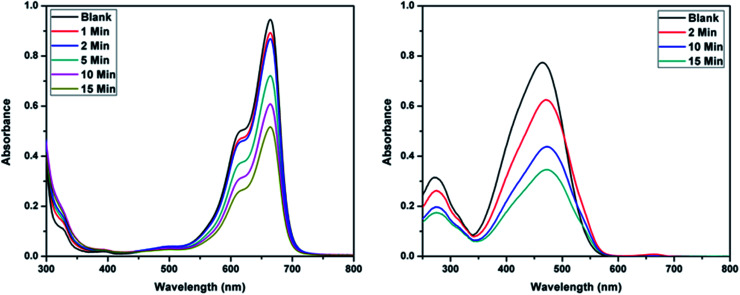
UV-visible absorbance spectra showing the adsorptive removal of (a) methylene blue and (b) methyl orange by CEr-Sol.

**Fig. 15:**
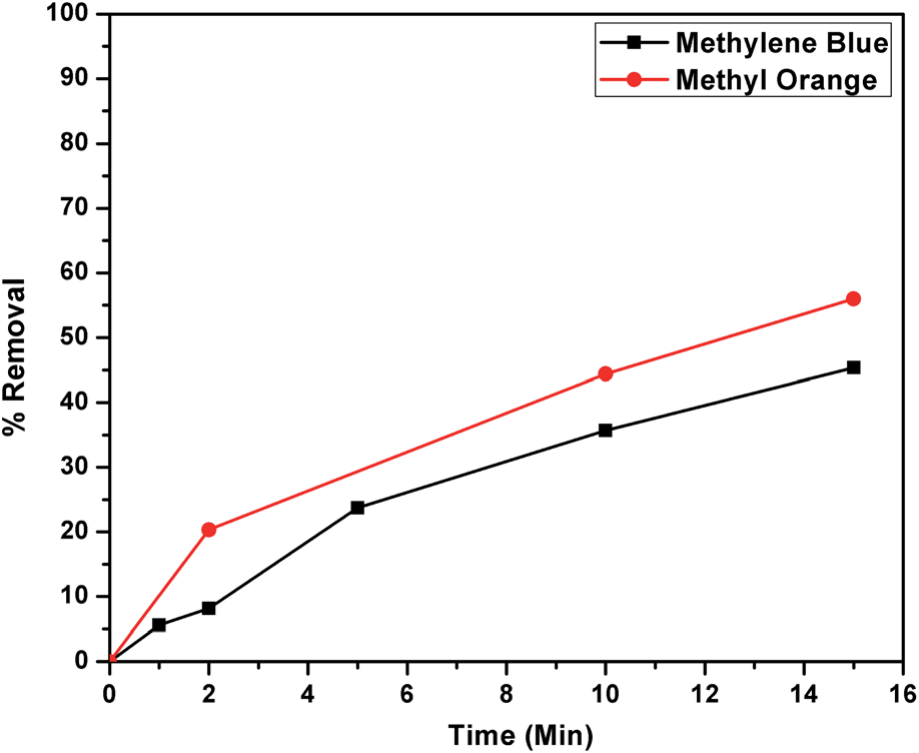
Removal percentages of methylene blue and methyl orange by CEr-Sol.

The higher selectivity of CEr-Sol towards Congo red (CR) was evaluated by carrying out adsorption analysis with a cationic dye methylene blue (MB) and another azo dye methyl orange (MO). The UV-visible absorption spectra for MB and MO adsorptions are shown in Fig. 14a and b, respectively. CEr-Sol shows adsorption towards both MB and MO to some extent. The percentage removal of MB and MO by CEr-Sol is shown in Fig. 15. CEr-Sol shows higher adsorption capacity towards MO than towards MB. Compared to the 85% removal of Congo red by CEr-Sol within 2 minutes, the percentage removal of MB and MO within the same time span is less.

**Fig. 16:**
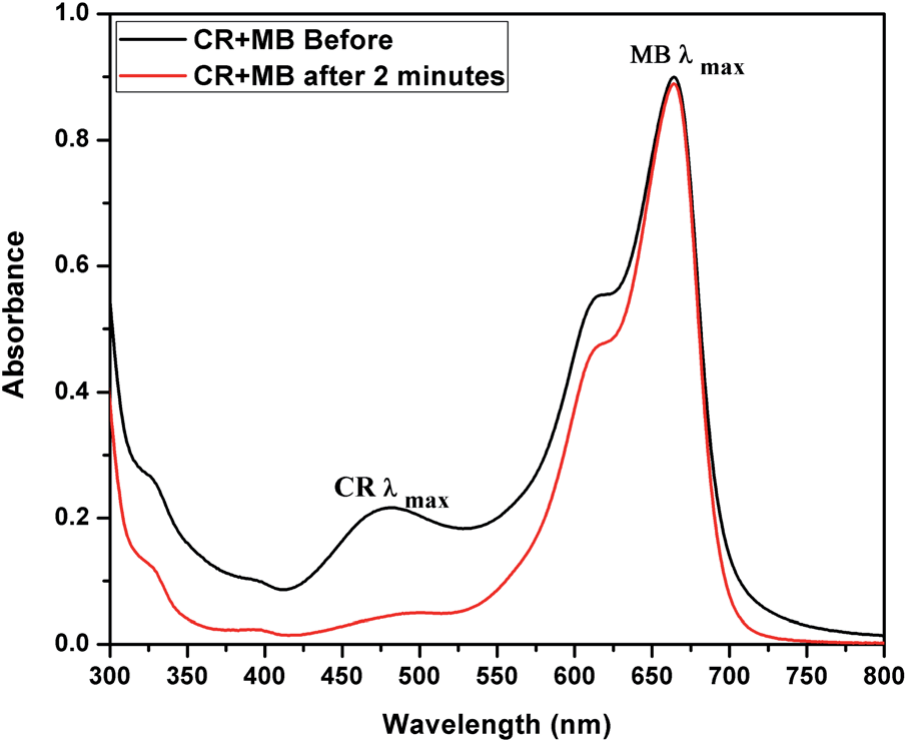
UV-visible absorbance spectrum showing the selective adsorption of Congo red by CEr-Sol from a mixed solution of Congo red and methylene blue.

The adsorption activity of CEr-Sol in a mixed dye solution of MB and CR was also studied using UV-visible absorption spectroscopy. UV-visible absorbance spectra of the mixed dye solution before and after introducing CEr-Sol are shown in Fig. 16. The initial dye solution exhibits two absorption maxima, one at 663 nm corresponding to MB and the other at ∼480 nm corresponding to CR, respectively. Two minutes after the introduction of CEr-Sol into the dye solution, a considerable reduction in the CR absorption band can be seen. At the same time, the MB absorption band around 663 nm is fully retained. Hence the selectivity and rapid adsorption rate of CEr-Sol towards CR even in the presence of other dyes are fully evident.

### Effect of pH on adsorption

**Fig. 17:**
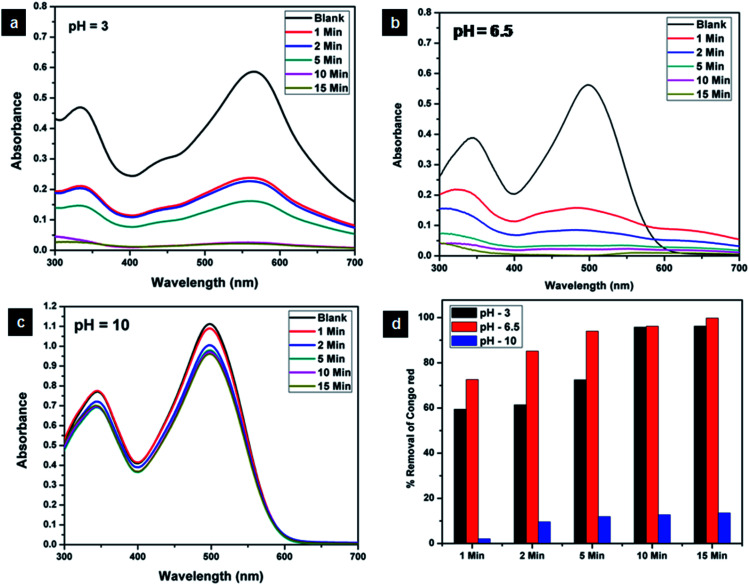
Effect of pH on the adsorptive removal of Congo red by CEr-Sol at (a) pH 3, (b) pH 6.5 and (c) pH 10 and (d) bar diagram showing the percentage removal at different time intervals.

The effect of pH on the adsorption of Congo red was evaluated by carrying out adsorption studies under pH conditions of 3, 6.5 and 10. The UV-visible spectra corresponding to the adsorption under three different pH conditions are shown in Fig. 17. On varying the pH of the Congo red solution, it was found that the colour of the solution turned dark blue at around pH 3 and showed a corresponding red shift in the absorption spectra. At the same time around pH 10, the red colour of the dye solution got more intense compared to that at the ambient pH of 6.5. It is evident from the adsorption studies that pH plays a crucial role in Congo red adsorption. CEr-Sol exhibited 99.75% removal of Congo red at the inherent pH (6.5) of the dye solution. At pH 3, CEr-sol showed a removal percentage of 96.24% which reduced to 13.54% at an alkaline pH of 10. Thus the inherent pH of the Congo red solution (6.5) was found to be the best environment for maximum adsorption by CEr-Sol. The pH of the point of zero charge (pH_PZC_) of CEr-Sol determined by the pH drift method was 2.16. The role of the pH of Cogo red solution and pH_PZC_ of the adsorbent in the adsorption mechanism is discussed in detail in the coming sections.

**Fig. 18:**
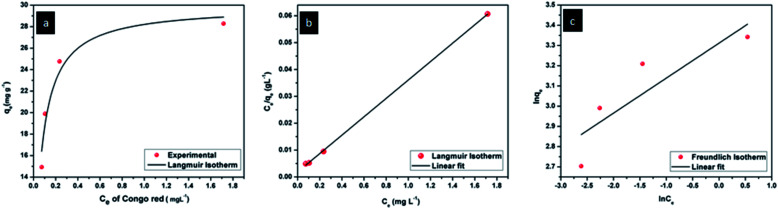
(a) Langmuir isotherm, (b) linearized Langmuir and (c) linearized Freundlich isotherms of CEr-Sol.

From all the above results, it is clear that CEr-Sol is the most superior and fastest adsorbent among the four variants of CeO_2_ samples synthesized. Adsorption isotherms of CEr-Sol are shown in Fig. 18.

Adsorption isotherm analysis can help to calculate the maximum adsorption capacity of adsorbents towards a particular species. The adsorbent concentration was optimized at first (1 g L^−1^). For the optimized CEr-Sol concentration, the amount of Congo red was varied (10, 15, 20, 25 and 30 mg L^−1^) and Langmuir and Freundlich adsorption isotherms were plotted. The Langmuir adsorption isotherm model can account for homogeneous systems. According to the Langmuir adsorption model
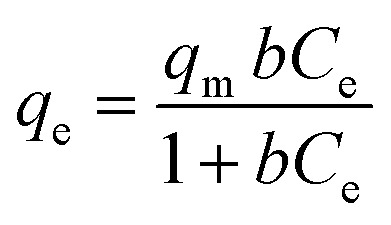
where *q*_e_ is the amount of dye adsorbed at equilibrium, *q*_m_ is the maximum amount of dye adsorbed per unit weight of the adsorbent (mg g^−1^) and *b* is the Langmuir adsorption isotherm constant.^59^

The Langmuir adsorption isotherm and linearized Langmuir isotherm of CEr-Sol are shown in Fig. 18a and b. The maximum amount of dye adsorbed per unit weight of CEr-SOl, *q*_m_, is found to be 29.19 mg g^−1^. It corresponds to the complete monolayer coverage of the CEr-Sol surface. The experimental data were found to fit well with the Langmuir model with a correlation coefficient *R*^2^ of 0.9996. The value of *b* is found to be 18.52 L mg^−1^ and *b* is a measure of affinity between the adsorbent and adsorbate. The Freundlich model can account for multilayer and non-equivalent adsorption sites. According to the Freundlich adsorption model
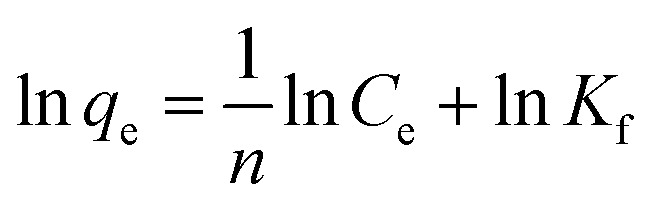
here *K*_f_ and *n* are Freundlich adsorption isotherm constants and *n* is the measure of the heterogeneity of the system.^59^ The linear fitting analysis of the experimental data with the Freundlich model as shown in Fig. 18c shows a correlation coefficient *R*^2^ of 0.6447. The *K*_f_ and *n* values are 27.428 and 5.77, respectively. From the *R*^2^ values, it is obvious that the Congo red adsorption on CEr-Sol follows the Langmuir model rather than the Freundlich one.

In the case of the Langmuir adsorption isotherm, the affinity between the adsorbent and adsorbate can be quantified by calculating the dimensionless separation factor *R*_L_ which is given by the equation
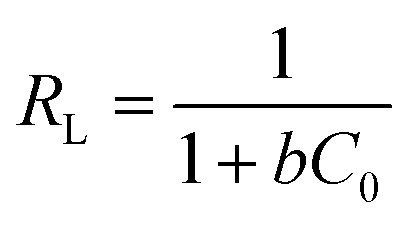
here *C*_0_ is the highest initial adsorbate concentration.^59^ Depending on the value of *R*_L_, adsorption can be classified into favourable and unfavourable adsorption. For favourable adsorption 0 < *R*_L_ < 1 and for unfavourable adsorption *R*_L_ > 1 or *R*_L_ = 1. For Congo red adsorption by CEr-Sol, we have obtained an *R*_L_ value of 0.0018. From the obtained *R*-value a favourable parameter *K*_*C*_0__ can be derived.
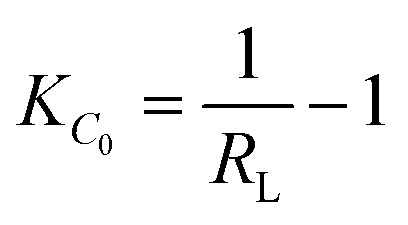


Linear isotherms will have a *K*_*C*_0__ value equal to 1. If *K*_*C*_0__ is in between 1 and 10 the adsorption is considered favourable. A *K*_*C*_0__ value higher than 10 denotes a spontaneous and highly favourable adsorption isotherm. The derived value of *K*_*C*_0__ for CEr-Sol is 557.65 and it indicated the highly favourable nature of its adsorption isotherm.

### Kinetics of Congo red adsorption on CEr-Sol

**Fig. 19:**
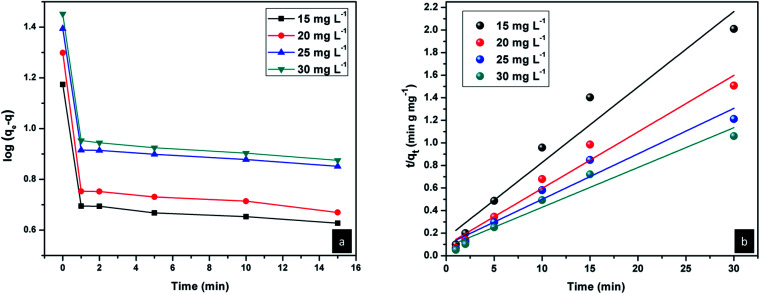
(a) Pseudo first order and (b) pseudo second order kinetics plots of CEr-Sol.

The rate of adsorption of Congo red by CEr-Sol can be determined from kinetics studies. Here we have considered pseudo first order and pseudo second order models for kinetics studies as shown in Fig. 19. According to the pseudo first order adsorption,
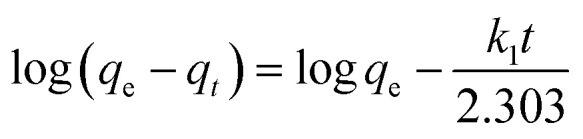
where *q*_e_ is the amount of Congo red adsorbed at equilibrium and *q*_*t*_ is the amount adsorbed during various time intervals. Here *t* is the time in minutes and *k*_1_ is the pseudo first order rate constant.^43^

In the case of pseudo second order adsorption,
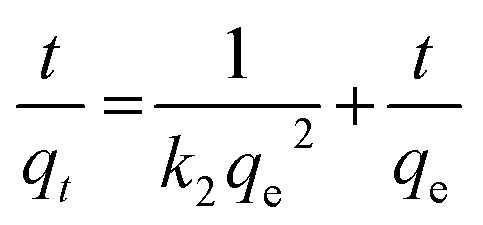
where *k*_2_ is the pseudo second order rate constant.^43^ Both the pseudo first-order and second-order kinetics models were applied to the Congo red adsorption by CEr-Sol and the model which fits best for the experimental data was identified from the linear regression correlation coefficient *R*^2^ values. From the linear regression analysis, it was observed that the experimental results fit well with pseudo second order adsorption. The pseudo second order rate constant *k*_2_, experimental and calculated values of *q*_e_ and *R*^2^ values at different concentrations of Congo red are given in Table 4. *R*^2^ values above 0.944 indicate the best fit of experimental data.

**Table 4:** Pseudo second order kinetics parameters for the selective adsorption of Congo red by CEr-Sol

Congo red concentration (mg L^−1^)	*k*_2_ (g mg^−1^ min^−1^)	*q*_e_ calculated (mg g^−1^)	*q*_e_ experimental (mg g^−1^)	*R* ^2^
15	2.84 × 10^−2^	14.95	14.93	0.9442
20	2.64 × 10^−2^	19.95	19.89	0.9649
25	1.68 × 10^−2^	24.81	24.76	0.9425
30	1.63 × 10^−2^	28.34	28.28	0.9534

## Mechanism of selective adsorption of Congo red

A thorough investigation of the adsorption mechanism was required to understand the selectivity and enhanced adsorption activity of the prepared samples towards Congo red. While evaluating the adsorption mechanism, several factors such as surface area, porosity, pH, electrostatic interaction between the adsorbent surface and dye molecules, weak interactions such as hydrogen bonding and coordination effects should be considered. The surface area and pore size distributions of the developed samples are already given in Table 2. Among the four CeO_2_ samples CEr-Sol has the highest surface area and the smallest pore size distribution (4.8 nm). Adsorption capacity and surface area are directly related. The molecular size of Congo red is 2.62 nm which can fit perfectly into the CEr-Sol pores, but the molecular sizes of methyl orange and methylene blue are 1.2 and 1.43 nm, respectively, which are too small to fit into the pores.^60^ Thus surface area enhancement and pore size regulation by doping Er^3+^ into the CeO_2_ lattice can influence the adsorption capacity to a certain extent. It is noted that the electrostatic interaction between the CeO_2_ surface and dye molecules can affect the adsorption capacity. Hence the adsorption activities of CEr-Sol using both cationic (methylene blue) and anionic (Congo red and methyl orange) dyes are also evaluated. Within 2 minutes, 85.1% of Congo red and 20.3% of methyl orange were removed by CEr-Sol. However, only 8.1% of the cationic dye methylene blue was removed in 2 minutes by CEr-Sol. Even though CEr-Sol has more affinity towards anionic dyes than towards cationic dyes, the surface charge on CeO_2_ is not the only crucial determining factor of adsorption here. If the surface charge was the only determining factor, CEr-Sol would have adsorbed both anionic dye species to the same extent. A combined effect of pH and adsorbent surface charge emerges during the adsorption of Congo red by CEr-Sol. Here the pH_PZC_ of CEr-Sol was found to be 2.16 (ESI Fig. S1†). Since Congo red is a dipolar molecule, it exists in anionic form in neutral and alkaline pH and in cationic form in acidic pH. From the pH_PZC_ value, we know that at pH > 2.16, the CEr-Sol surface is negatively charged and at pH < 2.16, the CEr-Sol surface is positively charged. While considering the possible electrostatic interactions between Congo red and the CEr-Sol surface, it can be seen that at neutral pH and pH above 7, the adsorbent surface and the Congo red molecules are negatively charged. Thus the mutual repulsion between the adsorbent and adsorbate is responsible for the reduced adsorption under alkaline conditions. Again below pH 2.16, we have both adsorbent and adsorbate as positively charged species and their mutual repulsion tends to decrease the extent of adsorption. This electrostatic repulsion is one of the reasons for the reduced adsorption under acidic conditions. At the same time, a pH in between 2.16 and 7 can give rise to positively charged Congo red species and a negatively charged CEr-Sol surface, enhancing the electrostatic attraction between Congo red and CEr-Sol. Thus the pH range in between 2.16 and 7 electrostatically favours Congo red adsorption on the CEr-Sol surface. Further beyond the electrostatic interactions, the deciding factor in this case is hydrogen bonding. Adsorbed water molecules and surface functional groups on the CeO_2_ surface can often form hydrogen bonds with the functional groups present on the dye molecules. Here the surface basic sites are significant in controlling the adsorption mechanism because the extent of hydrogen bonding depends on surface basicity. Hence further investigation is exclusively dedicated to the surface basicity controlled selective adsorption of Congo red by CeO_2_.

### Selective adsorption and surface basicity

**Fig. 20:**
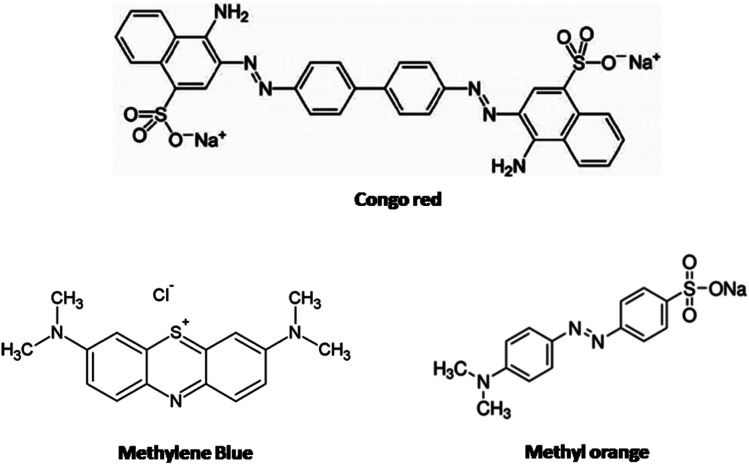
Structures of dye molecules under investigation.

**Fig. 21:**
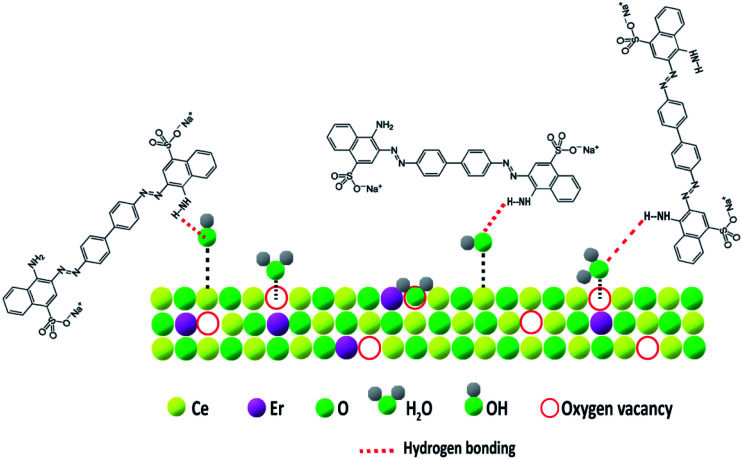
Selective adsorption mechanism of Congo red by CEr-Sol.

The type and nature of surface basic sites may be different in different adsorbent materials. Here on the CeO_2_ surface, oxygen vacancies are the major basic sites. The basic nature of oxygen vacancies was explained earlier. The doping of low valence ions such as Er^3+^ into the CeO_2_ lattice significantly increased the number of oxygen vacancies and thereby the amount of basic sites.^58^ These oxygen vacancies have a strong affinity for moisture.^53^ The presence of adsorbed moisture is evident from the FT-IR spectra. The adsorbed water molecules can form hydrogen bonds with solvent water which ensures better dispersion of the adsorbent in water. Also, many of the adsorbed water molecules dissociate near oxygen vacancies to form surface-active hydroxyl groups.^61^ The possibility of hydrogen bonding increases with an increase in surface basicity. Now the selectivity of CEr-Sol towards Congo red can be explained based on hydrogen bonding. The structures of Congo red, methylene blue and methyl orange are shown in Fig. 20. From the chemical structures, it is evident that only Congo red has –NH_2_ functional groups present in its structure which are capable of forming hydrogen bonds with the adsorbed water molecules and hydroxyl basic sites on the CeO_2_ surface. At the same time, methylene blue and methyl orange lack amino groups that are capable of forming hydrogen bonds. Thus hydrogen bonding can account for the selective adsorption of Congo red by CeO_2_. The proposed adsorption mechanism is illustrated in Fig. 21.

Since CEr-Sol possesses the highest amount of basic sites specifically medium strength and strong basic sites, a corresponding improvement in the adsorption capacity is observed. So the surface basicity enhancement by Er^3+^ doping into the CeO_2_ lattice increases hydrogen bonding and thereby the selective adsorption of Congo red. These studies revealed that surface basicity can be used as an effective tool for tuning the adsorption capacity and selectivity of CeO_2_ towards Congo red. Optimising surface basicity can thus lead to the fabrication of the best adsorbent version of CeO_2_ for environmental remediation.

### Reusability tests

**Fig. 22:**
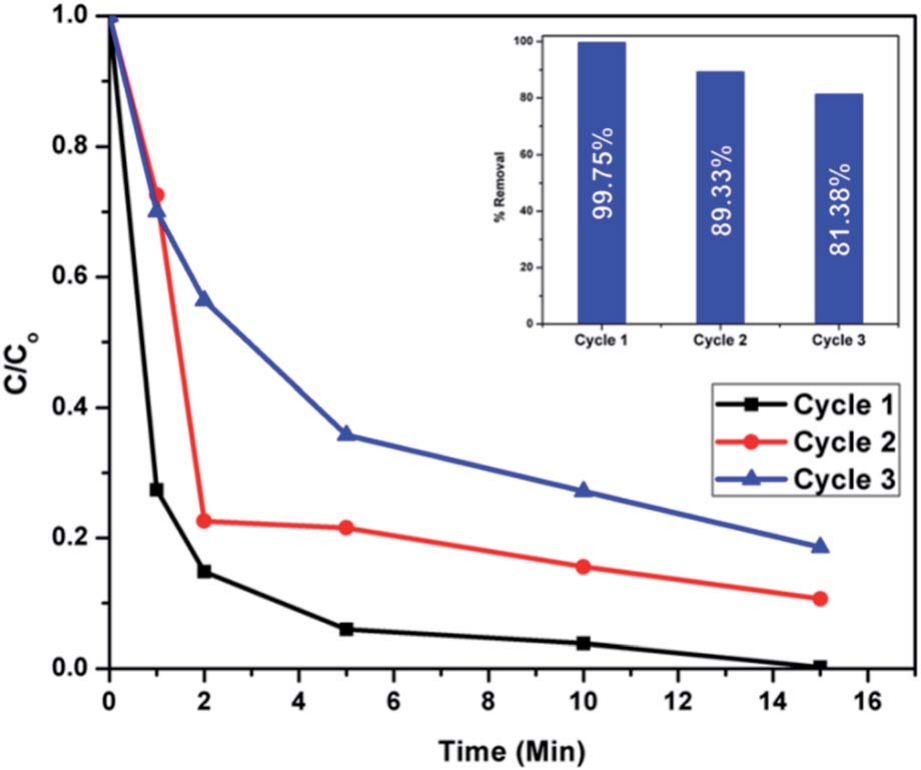
Adsorption rates of regenerated CEr-Sol samples in 3 successive cycles (the percentage removal is given in the inset).

Recyclability is an important attribute in sustainable environmental remediation. After washing several times with distilled water the regenerated adsorbents were dried and calcined at 500 °C for 2 hours. Adsorption studies were again carried out using the regenerated adsorbents for two more adsorption/regeneration cycles. As shown in Fig. 22, the recyclability tests show that the percentage removal of Congo red is 89.33% in the second cycle and 81.38% in the third cycle. Better desorption and recycling techniques which can preserve the surface active sites and thereby the adsorption efficiency of regenerated adsorbents should be further investigated.

## Conclusions

Nanocrystalline mesoporous CeO_2_ and Er^3+^ doped CeO_2_ samples were synthesized using sol–gel and sol–hydrothermal methods. The selective and rapid adsorption ability of these nanocrystalline mesoporous CeO_2_ samples towards organic pollutants such as Congo red, methyl orange and methylene blue was investigated in detail. From the adsorption experiments, CEr-Sol was found to be the most efficient and highly selective adsorbent towards Congo red. All these studies revealed that CEr-Sol has the highest selectivity and efficiency for Congo red adsorption. CEr-Sol is capable of removing 85.16% of Congo red within 2 minutes and 99.75% removal is observed in 15 minutes. The rapid and selective adsorption mechanism of CEr-Sol was further investigated in detail. Kinetics of the adsorption process was also studied and pseudo second-order kinetics was assigned to it. The main factors controlling the selectivity and adsorption ability are surface area, porosity, electrostatic interactions, surface active sites and hydrogen bonding. Among these, the presence of strong surface basic sites and hydrogen bonding are the crucial factors responsible for the selective and rapid adsorption of Congo red by CEr-Sol. From the surface basicity measurements, the enhancement of adsorption efficiency along with an increase in surface basic sites is evident. The basic mechanism behind the rapid and selective adsorptive removal of Congo red by CEr-Sol is the formation of hydrogen bonds, formed either by the surface hydroxyls or adsorbed water molecules with NH_2_ groups present exclusively on Congo red. Oxygen vacancies are the prominent basic sites present on the CeO_2_ surface. The enhanced number of oxygen vacancies generated upon Er^3+^ doping into the CeO_2_ lattice plays a significant role in selective adsorption. These oxygen vacancies have a higher affinity for moisture adsorption and the adsorbed water molecules can selectively form hydrogen bonds with the NH_2_ groups present on Congo red molecules. Since functional groups capable of hydrogen bond formation are absent in methylene blue and methyl orange, the extent of adsorption of these dye molecules on CEr-Sol is comparatively small. This work establishes the possibility of surface basicity mediated enhancement of the selectivity and efficiency of Congo red adsorption by CeO_2_. Thus surface basicity can be effectively used to tune the selective adsorption capacity of adsorbents towards pollutants.

## Conflicts of interest

There are no conflicts to declare.

## Supplementary Material

NA-003-D1NA00412C-s001
