# Supplementary material for: Surface basicity mediated rapid and selective adsorptive removal of Congo red over nanocrystalline mesoporous CeO2
Source: Nanoscale Adv. 2021 Sep 21;3(23):6704–18. doi: 10.1039/d1na00412c (PMC9419567; doi:10.1039/d1na00412c)
Supplement: NA-003-D1NA00412C-s001 [file NA-003-D1NA00412C-s001.pdf]

Electronic Supplementary Material (ESI) for Nanoscale Advances.
This journal is © The Royal Society of Chemistry 2021

Electronic Supplementary Information

Surface basicity mediated rapid and selective adsorptive removal of Congo red over nanocrystalline mesoporous CeO_2

Deepak Joshy [a], Seena Chakko [a], Yahya A. Ismail[a], Pradeepan Periyat*[a, b]

[a]Department of Chemistry, University of Calicut, Kerala, India-673635.

[b]Department of Environmental Studies, Kannur University, Kerala, India

E-mail:pperiyat@uoc.ac.in/pperiyat@kannuruniv.ac.in

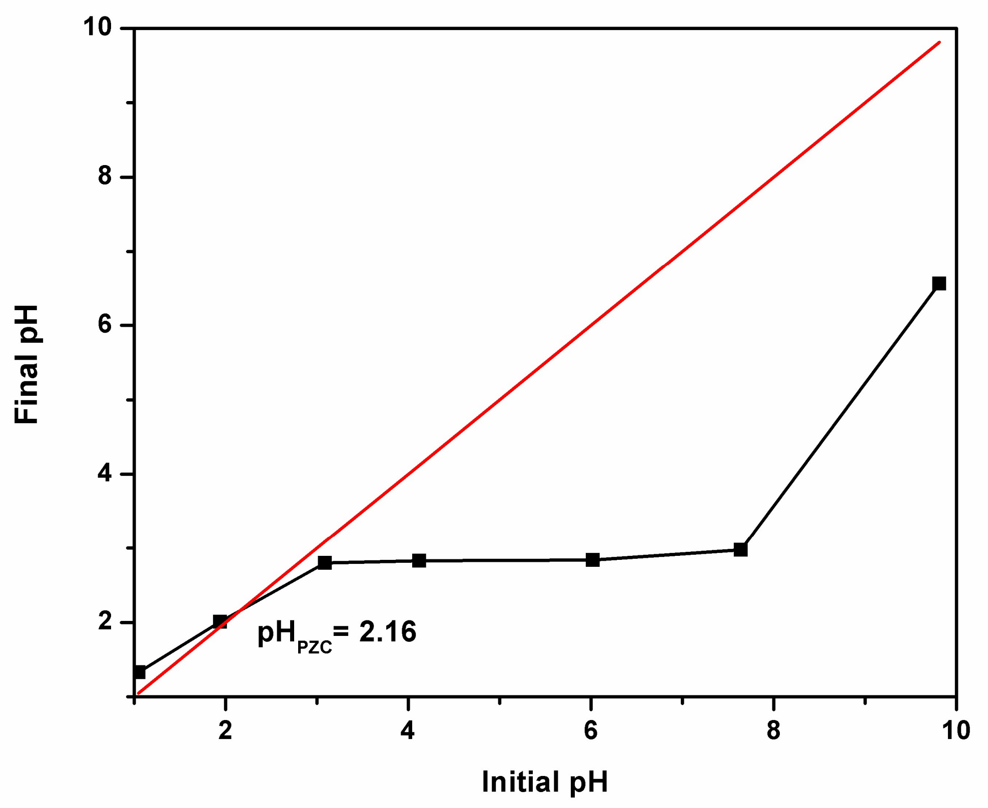

Fig. S1 The plot showing the determination of pH_{PZC} by pH drift method.
